# Unveiling the Silent Ductus: A Classical Presentation of Patent Ductus Arteriosus in an Asymptomatic 23-Year-Old Male

**DOI:** 10.7759/cureus.42678

**Published:** 2023-07-30

**Authors:** Poornima J Charpuria, Narendranath R Ganampet, Shresta M Kurian, Dirgha Patel, Praver C Chemudupati Parven, Mihirkumar P Parmar, Vishal Venugopal

**Affiliations:** 1 Internal Medicine, Osmania Medical College, Hyderabad, IND; 2 Internal Medicine, Chigateri General Hospital, Davanagere, IND; 3 Internal Medicine, Kamineni Academy of Medical Science And Research Centre, Hyderabad, IND; 4 Internal Medicine, Chigateri General Hospital, Davangere, IND; 5 Internal Medicine, Chalmeda Anand Rao Institute of Medical Sciences, Karimnagar, IND; 6 Internal Medicine, Baroda Medical College, Vadodara, IND; 7 Internal Medicine, Gandhi Medical College and Hospital, Hyderabad, IND; 8 Internal Medicine, Gujarat Medical Education & Research Society Medical College, Mehsana, IND; 9 Internal Medicine, Bhaarath Medical College and Hospital, Chennai, IND

**Keywords:** murmur, heart disease, asymptomatic, adult patient, patent ductus arteriosus

## Abstract

The patent ductus arteriosus (PDA) refers to the persistence of a connection between the descending aorta distal to the left subclavian artery and the pulmonary trunk beyond fetal life. Adult congenital heart disease is a rare condition, with asymptomatic cases being particularly uncommon. The following report presents the case of a young adult male, aged 23, who was discovered to possess a patent ductus arteriosus in an incidental manner. The patient presented with an acute chest complaint and was found to be asymptomatic upon examination at the hospital. Based on the preliminary medical information provided, a tentative diagnosis of a ventricular septal defect was established. However, a comprehensive echocardiographic examination revealed the presence of a patent ductus arteriosus (PDA).

## Introduction

The ductus arteriosus (DA) is a vascular connection that establishes a connection between the descending thoracic aorta and the pulmonary artery (PA) trunk. The beginning of the ductus arteriosus (DA) in the pulmonary artery (PA) is in the upper part of the PA, close to where the left PA splits into two and near where the left PA starts [1]. Usually, there are a lot of life-threatening symptoms associated with patent DA. There has been some recent focus on the silent ductus arteriosus, a small ductus that is not typically detected through auscultation but may be discovered incidentally during echocardiography conducted for other reasons. It is worth noting that these occurrences are uncommon, with estimates suggesting a prevalence of approximately one in every 500 to 1,000 individuals [2].

As a result of postnatal changes, there is a reversal of shunt leading to a left-to-right shunt due to the higher systemic circulation pressure compared to the pulmonary pressure. As a result of the elevated pressure gradient observed between the aorta and the pulmonary artery, it is common for the blood volume passing through the shunt to be increased. The aforementioned condition results in a volume overload on the right side of the heart, leading to the development of pulmonary hypertension characterized by increased resistance [3]. The primary complications associated with patent ductus arteriosus (PDA) include left cardiac insufficiency and infective endarteritis. The presence of a left-to-right shunt determines left ventricular overload (LV). Initially, the LV becomes hypercontractile and subsequently dilates and becomes insufficient during the course of the condition. The dilation of the left atrium (LA) is also caused by volume overload. Infective endarteritis of the ductus arteriosus is commonly known as a fatal complication of patent ductus arteriosus prior to the implementation of antibiotherapy. The administration of antibiotics has brought about a notable alteration in the frequency and prognosis of endarteritis among patients with PDA. However, it continues to be a potent complication even in cases where PDA is asymptomatic [4,5].

## Case presentation

**Figure 1:**
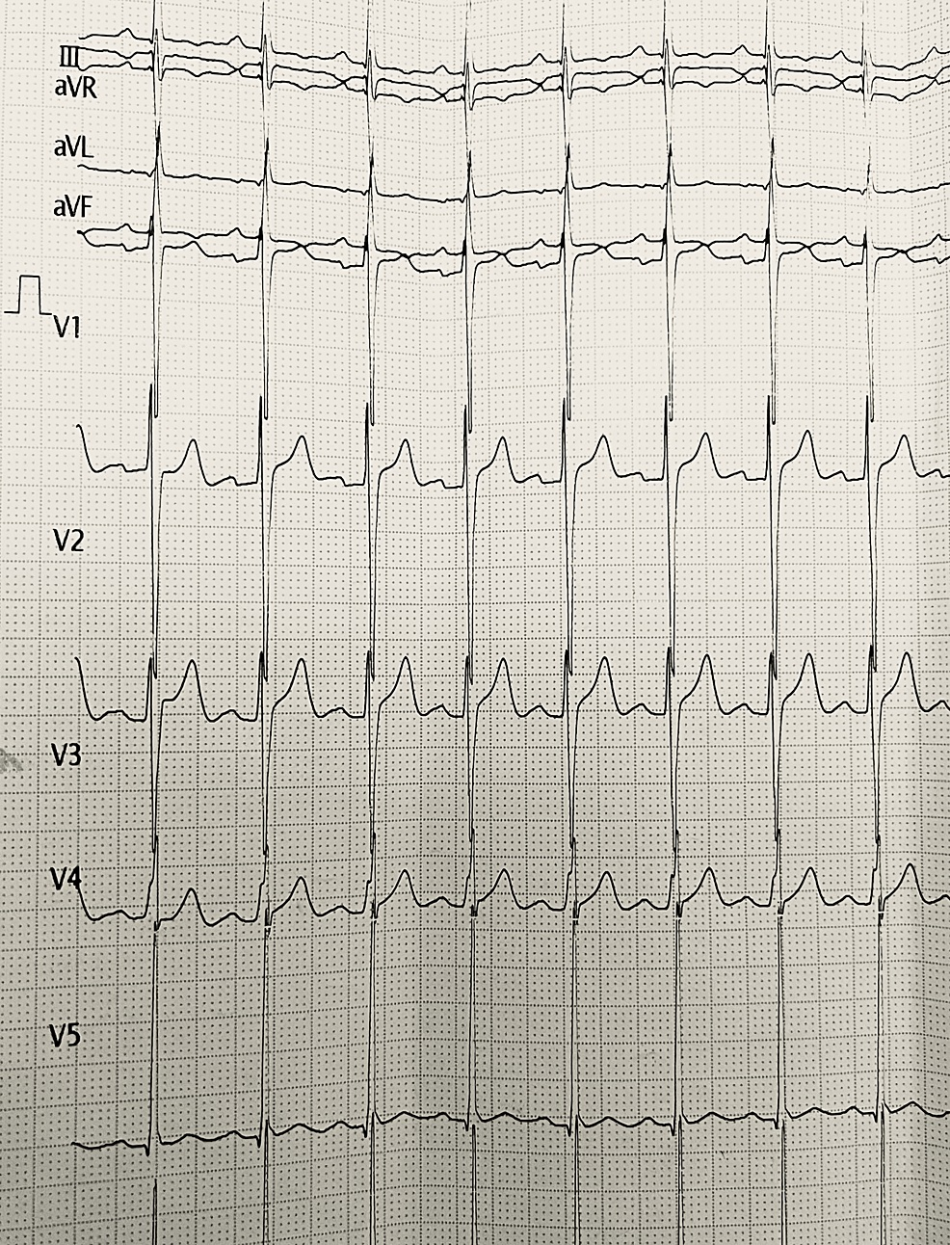
Presence of T wave inversions as well as hyperacute T waves Anatomical correlations of leads in a conventional 12-lead ECG: inferior surface of the heart (II, III, and aVF); anterior surface (V1 to V4); I, aVL, V5, and V6 refer to the lateral surface. Right atrium V1 and aVR refer to the left ventricle's cavity.

A 23-year-old man presented to the hospital with the chief complaint of chest pain that he had been experiencing for the past day. The pain was not radiating in nature, and the patient had a history of similar episodes dating back to childhood. During the examination, it was found that the patient's jugular venous pressure was high, his blood pressure was around 176/90 mmHg on the right arm brachial artery, and his pulse was around 112 beats per minute on the right arm radial artery at the wrist. The patient underwent auscultation, which revealed the presence of a pansystolic murmur. Subsequently, an electrocardiogram (ECG) was conducted, which revealed the presence of T wave inversions as well as hyperacute T waves (Figure 1).

A presumptive diagnosis of ventricular septal defect was made on the basis of a pansystolic murmur, and the patient was admitted to the inpatient ward. Congenital and structural echocardiography revealed adult congenital heart disease (ACHD) with left atrial and left ventricular dilation, no pulmonary arterial hypertension, and a 6 mm patent ductus arteriosus with a left to right shunt with a 127/52 mmHg gradient with normal biventricular function and a normal aortic arch. Laboratory results for serum urea, serum creatinine, serum sodium, serum potassium, and serum chloride were within normal limits (Table 1).

**Table 1:** Laboratory results ISE - ion selective electrode (ISE); In contrast to "concentration in the plasma (mmol/L)", direct ISE really measures the electrolyte activity in the plasma water (mmol/kg H2O).

Biochemistry		
Test parameter	Results	Reference range
Urea endpoint/colorimetric: urease	19.0 mg/dl	Male: 19-43 mg/dl; Female: 15–36 mg/dl
Serum creatinine two-point rate: creatinine aminohydrolase	1.1 mg/dl	Male: 0.8–1.5 mg/dl; Female: 0.7-1.2 mg/dl
Serum electrolytes (Na, K, and Cl)		
Test parameter	Results	Reference range
Serum sodium direct ISE: potentiometric	137.0 mmol/L	137-145 mmol
Serum potassium direct ISE: potentiometric	4.1 mmol/L	3.5-5.1 mmol/L
Serum chloride direct ISE: potentiometric	100.0 mmol/L	98-107 mmol/L

The treatment plan included medication such as injection of Xone 1 gm (ceftriaxone), injection of pantoprazole 1mg, injection of lasix 40 mg, tablet Montek LC (a combination of two: montelukast and levocetirizine), tablet Ultracet, and infusion of paracetamol were all part of the treatment plan. In addition, the patient was examined for any indications of pulmonary arterial hypertension, but those symptoms were not found to be present.

## Discussion

The patient was a 23-year-old male who presented to the hospital with chest pain lasting for a day. The pain was not radiating, and the patient had a history of similar episodes since childhood. On examination, raised jugular venous pressure, elevated blood pressure, and a high pulse rate were observed. Auscultation revealed a pansystolic murmur. An ECG showed T wave inversions and hyperacute T waves. A provisional diagnosis of ventricular septal defect was made based on the murmur, and the patient was admitted to the inpatient ward. Further investigation of congenital and structural echocardiography revealed congenital heart disease with left atrial and left ventricular dilation, a 6 mm patent ductus arteriosus with a left to right shunt, and normal biventricular function and aortic arch.

PDA affects 5-12% of all congenital cardiac defects and affects women twice as frequently as men. The expected annual mortality rate for adults with untreated PDA is 1.8% [6, 7]. Also, 20-50% of neonates born before 32 weeks of gestation and up to 60% of neonates born before 29 weeks of gestation are born with patent ductus arteriosus [8, 9]. PDA is typically identified inadvertently during a physical examination or echocardiographic screening in adults. Although it may be asymptomatic, PDA is defined by a persistent murmur at the upper left sternal border [7, 10].

Silent PDA that has been tolerated for years may become clinically important in the presence of acquired conditions such as the onset of chronic obstructive pulmonary disease or signs of valvular or ischemic heart disease [6, 7, 10]. Some people can develop endocarditis, congestive heart failure, pulmonary hypertension, signs of right or left ventricular volume overload, or recurrent pneumonia. Pulmonary artery aneurysms, another rare condition, could be caused by structural cardiac defects, particularly congenital heart illnesses such as PDA [6, 7, 10]. Because of the likelihood of spontaneous closure of PDA, the option to treat it remains debatable. Conservative therapy, pharmaceutical therapies, surgical ligation, and a transcatheter approach to ductal closure are all alternatives for hemodynamically significant PDA (hsPDA). However, agreement on PDA management strategies is still elusive. When all other medicinal therapies have failed or are contraindicated, surgical ligation is frequently explored [11].

In our case, we have given medications such as ceftriaxone, pantoprazole, lasix, Montek LC, Ultracet, and paracetamol infusion. The patient was also monitored for signs of pulmonary arterial hypertension, which were not present. Laboratory results were normal for serum urea, creatinine, sodium, potassium, and chloride levels, which suggests that there were no other underlying diseases. The diagnostic efficacy of the clinical examination for PDA is limited. Thus, echocardiography, particularly Doppler echocardiography, is an essential diagnostic tool for identifying PDA [12]. According to published research, transesophageal echocardiography is more sensitive than transthoracic echocardiography in identifying adult PDA. Magnetic resonance imaging (MRI) or multi-slice computer tomography (MSCT) can then be used to confirm the diagnosis [13]. The size, shape, calcification, and friability of the PDA heavily influence the selection of surgical repair methods [14].

The pansystolic murmur in our patient's situation resulted in a false-positive diagnosis of VSD. Due to increased cardiac output, tachycardia and bounding peripheral pulses develop. The cause of increased pulse pressure (>30 mmHg) is thought to be a combination of decreased diastolic blood pressure from the run-off and a slight rise in systolic blood pressure to compensate for the decrease in distal blood flow caused by the PDA run-off during diastole. Even with the most recent diagnoses, undetected PDA still has a significant frequency in adult populations. Physicians are frequently taken in by its various clinical manifestations. A pulmonary trunk aneurysm that causes an excessive amount of blood to flow into the heart during surgery raises the possibility of an undiagnosed PDA.

## Conclusions

It is recommended that patients with continuous murmurs in the precordial region undergo an echocardiographic evaluation. In cases where the echocardiogram is inconclusive, additional imaging modalities may be considered. We acknowledge the significance of surgical intervention in addressing a patent ductus arteriosus, even if it is asymptomatic. However, we advise against the notion that all patients with this condition should undergo routine antibiotic prophylaxis and surgical ligation. It is important to carefully evaluate each patient's individual circumstances before making any treatment decisions.

## References

[1] Schneider DJ, Moore JW (2006). Patent ductus arteriosus. Circulation.

[2] Lloyd TR, Beekman RH 3rd (1994). Clinically silent patent ductus arteriosus. Am Heart J.

[3] Wung JT, James LS, Kilchevsky E, James E (1985). Management of infants with severe respiratory failure and persistence of the fetal circulation, without hyperventilation. Pediatrics.

[4] Cruz-González I, Martín-Herrero F, Sánchez JL (2006). Infective endarteritis in patent ductus arteriosus and septic pulmonary embolism (Article in Spanish). Rev Esp Cardiol.

[5] Ozkokeli M, Ates M, Uslu N, Akcar M (2004). Pulmonary and aortic valve endocarditis in an adult patient with silent patent ductus arteriosus. Jpn Heart J.

[6] Cassidy HD, Cassidy LA, Blackshear JL (2009). Incidental discovery of a patent ductus arteriosus in adults. J Am Board Fam Med.

[7] Sa-Kong H, Seol SH, No TH, Park DH, Jeong NR, Jeong SJ, Kim DI (2017). Huge idiopathic pulmonary artery aneurysm. Radiol Case Rep.

[8] Sellmer A, Bjerre JV, Schmidt MR (2013). Morbidity and mortality in preterm neonates with patent ductus arteriosus on day 3. Arch Dis Child Fetal Neonatal Ed.

[9] Hamrick SE, Hansmann G (2010). Patent ductus arteriosus of the preterm infant. Pediatrics.

[10] Navaratnarajah M, Mensah K, Balakrishnan M, Raja SG, Bahrami T (2011). Large patent ductus arteriosus in an adult complicated by pulmonary endarteritis and embolic lung abscess. Heart Int.

[11] Malviya MN, Ohlsson A, Shah SS (2013). Surgical versus medical treatment with cyclooxygenase inhibitors for symptomatic patent ductus arteriosus in preterm infants. Cochrane Database Syst Rev.

[12] Davis P, Turner-Gomes S, Cunningham K, Way C, Roberts R, Schmidt B (1995). Precision and accuracy of clinical and radiological signs in premature infants at risk of patent ductus arteriosus. Arch Pediatr Adolesc Med.

[13] Boyalla V, Putzu P, Dierckx R, Clark AL, Pellicori P (2015). Patent ductus arteriosus in older adults: incidental finding or relevant pathology?. J Am Geriatr Soc.

[14] Djukanovic BP, Micovic S, Stojanovic I, Unic-Stojanovic D, Birovljev S, Vukovic PM (2014). The current role of surgery in treating adult patients with patent ductus arteriosus. Congenit Heart Dis.

